# Open nephron-sparing surgery in renal tumors with normal contralateral kidney: A single centre experience of 8 years

**DOI:** 10.4103/0970-1591.30259

**Published:** 2007

**Authors:** N. P. Gupta, A. Kumar, A. K. Hemal, P. N. Dogra, A. Seth, R. Kumar

**Affiliations:** Department of Urology, All India Institute of Medical Sciences, New Delhi, India

**Keywords:** Nephron sparing surgery, open surgery, renal tumor

## Abstract

**Introduction:**

We present our eight-year experience with open nephron-sparing surgery (NSS) in renal tumors with contralateral normal kidney to assess its oncological efficacy and safety.

**Materials and Methods:**

Thirty-six patients undergoing open NSS for small localized renal tumors with normal contralateral kidney from January 1998 to August 2006 were studied regarding demographic, clinical and pathological characteristics along with long-term follow-up.

**Results:**

The mean age was 48.28 ± 9.5 years. The mean tumor size was 3.72 cm (range 1.5-6). The following surgeries were performed: Wedge resection-13, partial polar nephrectomy-15, segmental resection-eight. The following techniques were used for vascular control: clamping and cooling-eight, warm ischemia-12, a novel technique of serial encirclage-16. The mean warm ischemia time was 23.2 ± 3.2 min. The mean operating time was 190.07 ± 11.3 min. The mean estimated blood loss was 331 ± 17.4 ml. The majority of renal tumors were renal cell carcinoma (97.22%). There were no positive surgical margins. There were no major intraoperative and postoperative complications. The mean follow up was 52.1 months (range 4-80) with no case showing progression to renal insufficiency (defined as serum creatinine > 2 mg/dl). There was only one local recurrence. However, four distant metastases were reported. The five-year cancer-specific survival, recurrence-free survival and overall survival were 94.4%, 88.88% and 86.11% respectively.

**Conclusions:**

In patients with solitary, small localized, unilateral renal tumors with normal contralateral kidney, elective open NSS is feasible, safe and provides excellent long-term local control and oncological efficacy with functional benefits.

Radical nephrectomy (RN) has been considered the standard treatment for localized renal tumors with an anatomically and functionally normal contralateral kidney. The first nephron-sparing surgery (NSS) for a renal tumor was done by Czerny in 1887.[1] The initial indications of NSS for renal tumors were either absolute including a solitary kidney and bilateral renal tumors or relative, which include impending or existing renal failure.[2] Since then, interest in NSS has been growing in localized renal tumors with normal contralateral kidney due to several developments including advances in renal imaging, improved surgical techniques and methods to prevent ischemic renal injury, increasing number of incidentally detected small tumors due to better imaging and reduced complications due to better postoperative care including renal replacement therapy.[3] The various series have proved that NSS is safe, cost-effective and results in low morbidity.[4,5] Moreover, NSS has shown effective local control and comparable five-year cancer-specific survival rate for localized renal tumors with normal contralateral kidney, equivalent to radical nephrectomy.[6] Therefore, NSS is being increasingly used in these patients.[2,7] We present our eight-year experience with open NSS in small, localized, renal tumors with contralateral normal kidney to assess its feasibility, efficacy and long-term oncological efficacy in these patients.

## MATERIALS AND METHODS

This prospective study included a total of 36 consecutive patients undergoing elective open NSS for small, localized renal tumors (clinical stage, T1N0M0) with normal contralateral kidney between January 1998 and August 2006 in our institution. The patients with large or extensive tumors, suspected lymph node involvement, advanced age and significant illness were excluded from study. All patients underwent a detailed history, physical examination, a laboratory evaluation including serum creatinine, liver function tests and urine analysis. Radiographic testing was used to exclude locally advanced or metastatic disease including chest X-ray, ultrasound abdomen, contrast enhanced computed tomography (CECT)/ magnetic resonant imaging (MRI) abdomen.

### Surgical technique

The kidney was exposed using an extraperitoneal, extrapleural flank incision through the 11^th^ or 12^th^ rib bed. The kidney was mobilized within the Gerota's fascia leaving intact peri-renal fat around the tumor. The renal pedicle was dissected en-block and a vascular tape was encircled and kept ready for vascular clamping in selected cases. The patient was adequately hydrated and intravenous mannitol was given before arterial clamping and readministered after the vascular clamp was removed to promote brisk diuresis. When the anticipated time of arterial occlusion was more than 30 min, *in situ* renal hypothermia was used using peri-renal ice slush, to minimize ischemic injury to the kidney. Surgical hypothermia was established immediately after renal artery clamping and maintained for 10 min to decrease the core temperature before starting tumor resection. In 16 patients, we used a technique of serial encircling haemostatic sutures through the renal parenchyma proximal to the level of partial nephrectomy for renal cell carcinoma (RCC) to avoid cross clamping of the renal artery for polar lesions (unpublished data). We performed polar (apical, basilar) segmental nephrectomy, transverse and wedge resection, trying to remove at least 5 mm of normal tissue around the tumor, based on intraoperative palpation. In cases in which tumor was in close proximity to the pelvi-calyceal system, based on preoperative imaging study and there was anticipation of breech in the pelvi-calyceal system, methylene blue was instilled through a preplaced ureteric catheter to visualize breech of collecting system. This breech was then closed by absorbable sutures. We did not use intraoperative frozen section from tumor margin and intraoperative ultrasound. The renal defect was closed using approximating vicryl^®^ sutures over a bolster made of Gelfoam^®^/ Surgicel^®^/muscle.

The demographic, intraoperative and postoperative outcomes, pathological characteristics and follow-up were prospectively recorded. The patients were followed with an annual history, physical examination, measurement of serum calcium, alkaline phosphatase, liver and renal function tests. The ultrasound abdomen was performed at three months postoperatively and subsequently six-monthly for two years and then yearly thereafter, using CECT abdomen in case of abnormal ultrasound findings. The five-year overall survival, cancer-specific survival and recurrence-free survival were calculated using the Kaplan Meier method. The results were then analyzed statistically and presented as the mean value ± standard deviation.

## RESULTS

The demographic profile of the patients and tumor size are detailed in Table 1. 47.2% patients were detected as incidental renal masses. The mean tumor size was 3.72 cm (range: 1.5-6 cm). Table 2 summarizes the intraoperative and postoperative outcome. The novel technique of serial encircling sutures was used in 16 patients. The mean cold ischemia time, warm ischemia time and mean estimated blood loss were 37.1 ± 4.1 min (range: 31-47), 23.2 ± 3.2 min (range: 20-29) and 331 ± 17.4 ml respectively. There were no significant intraoperative complications except bleeding in three patients, which was controlled without any sequelae. The postoperative complications were also not significant, with no case reported of wound hernia.

**Table 1:** Patient demographics and tumor characteristics

Total no. of patients	36
Mean age (yrs)	48.28 ± 9.5
Male/female	26/10
**Clinical presentation (%)**	
Incidental	17(47.2)
Hematuria	4 (11.1)
Flank pain	15(41.6)
Side (right/left)	21/15
Mean tumor size in cm (range)	3.72 (1.5-6)
Upper pole tumor	10
Mid pole tumor	14
Lower pole tumor	12

**Table 2:** Intraoperative data and postoperative course along with complications

Wedge resection	13
Upper polar nephrectomy	8
Lower polar nephrectomy	7
Segmental resection	8
**Technique**	
Clamping and cooling	5
Warm ischemia	15
Serial encirclage	16
**Ischemia time in min (range)**	
Cold	37.1 1 ± (31-47)
Warm	23.2 ± 3.2 (20-29)
Mean operating time (min)	190.07 ± 11.3
Estimated blood loss (ml)	331 ± 17.4
Analgesic use (mg pethidine)	237.17 ± 21
Hospital stay (days)	5.5 ± 1.1
Weeks to normal activity	1.8 ± 0.3
**Intraoperative complications (%)**	
Bleeding	3 (8.3)
**Postoperative complications (%)**	5 (13.8)
Paralytic ileus	2
Transient decrease in renal function	0
Wound sepsis	2
Postop hemorrhage	1
Wound hernia	0

Table 3 summarizes the pathological and follow-up results. The majority (97.22%) of renal tumors were RCC and the clear cell was the predominant subtype of RCC (77.1%). There were no positive margins in any case. The majority of patients were in pT_1a_ N_0_ M0 (94.44%), having predominantly Grade 2 (41.6%).

**Table 3:** Pathological results and follow-up record of the patients

**Histopathology**	
**Renal cell carcinoma (%)**	35 (97.2)
Clear cell	28 (80)
Papillary	5 (14.3)
Chromophobe	2 (5.7)
**Oncocytoma (%)**	1 (2.7)
**Tumor stage** (%)	
pT1aN0MO	34 (94.4)
pT1bN0M0	2 (5.6)
**Tumor grade (%)**	
1	12 (33.3)
2	15 (41.6)
3	7 (19.4)
4	2 (5.5)
Mean follow-up in months (range)	52.1 (4-80)
No. of local recurrence	1
No. of distant metastases	4
5-Year cancer- specific survival rate (%)	94.44
5-Year recurrence-free survival rate (%)	88.88
5-Year overall survival rate (%)	86.11

The mean follow-up was 52.1 months (range: 4-80). Twenty-five (69.4%), 21 (58.3%) and 11 (30.5%) patients completed the follow-up of 36 months, 52.1 months and 60 months respectively. There was no case showing progression to renal insufficiency (defined as serum creatinine > 2 mg/dl). There was only one local recurrence in the ipsilateral kidney reported at 13 months of follow-up. This patient had a central tumor of 3.9 cm in the right kidney. There was no tumor spillage during the surgery. The histological report showed renal cell carcinoma (papillary), Grade 3 with negative margins. The patient underwent open right radical nephrectomy. The same patient developed lung metastases six months after surgery, highlighting the aggressive nature of the tumor.

**Figure 1:**
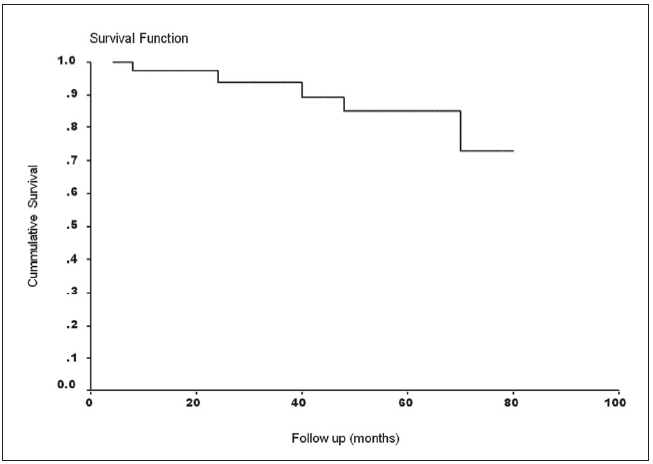
Kaplan-Meier survival curve for overall survival

**Figure 2:**
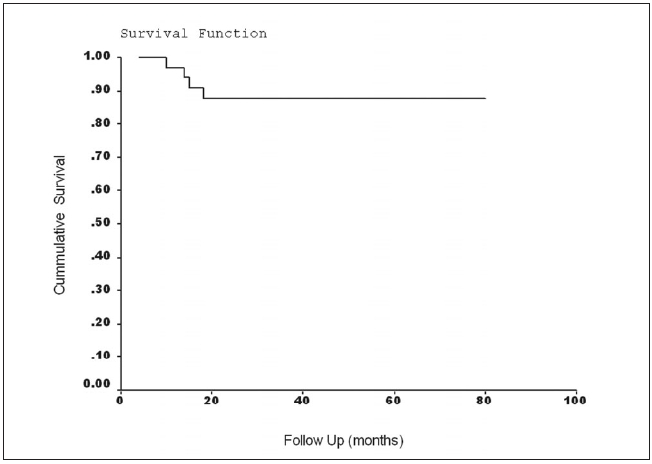
Kaplan-Meier survival curve for recurrence-free survival

There were four distant metastases (lung - three, bone - one) reported at follow-up of 13, 17, 19 and 26 months respectively. The number of events is too small to carry out multivariate analysis of various risk factors responsible for metastases. The tumor size in these patients was 6 cm, 5.2 cm, 3.9 cm and 4cm respectively. The tumors were centrally located in all patients. The histological types were RCC (clear cell with sarcomatoid differentiation), RCC (papillary), RCC (papillary) and RCC (clear cell with sarcomatoid differentiation) respectively. The histological grades were 4, 3, 3 and 4 respectively. One patient with lung metastasis and the other with bone metastasis died nine and 11 months after detection of metastases. There were three noncancer-related deaths (myocardial infarction - two, stroke - one). The five-year cancer-specific survival, recurrence- free survival and overall survival were 94.44, **88.88** and 86.11% respectively. Kaplan Meier survival curves for overall survival and recurrence-free survival are shown in Figure 1 and 2 respectively.

## DISCUSSION

After its introduction by Czerny[1] in 1887, NSS has evolved as a surgical option for localized renal tumors. The initial indications for NSS included patients with either an absolute indication like a solitary kidney, bilateral tumors or a relative indication like when the contralateral kidney is affected by a condition that threatens its future function.[3] Nephron-sparing surgery has been increasingly used in patients with small localized renal tumors (pT_1a_ N_0_ M_0_, size less than 4 cm) with normal contralateral kidney.[2,7–9] There is an expansion of the indications of use of NSS to pT_1b_ N_0_ M_0_ (size less than 7 cm) recently.[10,11] This can be explained on the basis that various reports have shown comparable local control and five-year cure rates of NSS, when compared with radical nephrectomy,[6] along with significant decrease in intraoperative and postoperative morbidity.[10,12]

The improved surgical techniques including better methods of hemostasis, preventing ischemic renal damage and meticulous dissection have significantly decreased intraoperative and postoperative complications. One of the major concerns during NSS is adequate hemostasis of the resected surface because various reports have shown intraoperative and delayed bleeding with secondary nephrectomy as its complications.[12,13] Various hemostatic methods like coronal sutures, fibrin glue, harmonic scalpel, lasers, electric current, dissecting clamp and a linear stapling device have been reported in the literature.[14] We have used various techniques of hemostasis including a simple, reliable and novel technique of serial encircling sutures (16 patients) wherever feasible, which avoids hilar clamping, thus causing no ischemic renal damage. This technique is inexpensive and can be easily practiced by all urologists (unpublished data). In our series, intraoperative (8.3%) and postoperative (2.7%) bleeding are comparable with other series.[12,13] Our mean warm ischemia time of 23.2 min is also comparable with other series (Gill *et al*[15] – 17.5 min, Ray *et al*[16] - 20 min).

The most common renal-related complications after NSS is urinary fistula, occurring in 6.5% patients (range - 1.4-17.4).[13] In our series, there was no case of urinary fistula. This can be attributed to our technique of using methylene blue injection through a preplaced ureteric catheter while excising tumor and subsequently, closing any defect in the collecting system intraoperatively, with absorbable sutures.

Another major concern with this approach has been the risk of local recurrence due to inadequate tumor excision or tumor multifocality, thus decreasing long-term survival. However, various studies have proved convincingly the long-term oncological efficacy of NSS in terms of local recurrence and five-year cure rates.[7,17,18] The local recurrence and five-year cancer-specific survival has been reported to be 0-7.3% and 89-98% respectively, in different series.[7–9,13,17] In our series, there were no positive margins. After a mean follow-up of 52.1 months (range: 4-80), there was only one local recurrence (2.7%). The high grade of the tumor and the possibility of undetected microscopic multifocal renal cell carcinoma, in view of papillary histology, can cause the local recurrence in this case. The five-year cancer-specific survival was 94.4%, thus comparable with other reported series. There were only four cases (11.1%) of distant metastases at 13, 17, 19 and 26 months respectively: lung - three, bone - one. There were only two (5.55%) cancer-related deaths and three (8.33%) noncancer-related deaths.

There are some definitive advantages of NSS in patients with normal contralateral kidneys. Firstly, there is a speculation that the advantage of maximal nephron preservation might decrease the risk of progression to chronic renal insufficiency and end stage renal disease. The recent studies by Lau *et al*[18] and McKiernan *et al*[19] have shown that RN resulted in higher cases of renal insufficiency (defined as increase in serum creatinine > 20 mg/L), when compared with NSS. In our series, no case showed progression to renal insufficiency (defined as increase in serum creatinine > 20 mg/dl) at a mean follow-up of 52.1 months, thus proving the functional benefits of NSS. Secondly, synchronous or metachronous bilateral involvement has been reported in 2-4% cases of sporadic RCC.[20] Therefore, in a patient of RCC with normal contralateral kidney, there is a possibility of involvement of the contralateral normal kidney in future by RCC. Therefore, we can preserve the maximum functional renal parenchyma in these patients by doing a nephron-sparing surgery.

## CONCLUSIONS

In patients with solitary, small localized, unilateral renal tumors with normal contralateral kidney, elective open NSS is an appropriate, safe and feasible surgical option, which provides excellent long-term oncological results with functional benefits.
